# Abnormal corneal epithelial maintenance in mice heterozygous for the micropinna microphthalmia mutation *Mp*

**DOI:** 10.1016/j.exer.2016.05.021

**Published:** 2016-08

**Authors:** Panagiotis Douvaras, Natalie J. Dorà, Richard L. Mort, Emily J. Lodge, Robert E. Hill, John D. West

**Affiliations:** a Genes and Development Group, Centre for Integrative Physiology, Clinical Sciences, University of Edinburgh Medical School, Hugh Robson Building, George Square, Edinburgh, EH8 9XD, UK; b Genes and Development Group, Centre for Integrative Physiology, Biomedical Sciences, University of Edinburgh Medical School, Hugh Robson Building, George Square, Edinburgh, EH8 9XD, UK; c Medical and Developmental Genetics Section, MRC Human Genetics Unit, MRC IGMM, University of Edinburgh, Western General Hospital, Crewe Road, Edinburgh, EH4 2XU, UK

**Keywords:** Corneal epithelium, Corneal homeostasis, Limbal epithelial stem cells, Limbus, Micropinna microphthalmia mutant, Mouse, Mp mutant

## Abstract

We investigated the corneal morphology of adult *Mp*/+ mice, which are heterozygous for the micropinna microphthalmia mutation, and identified several abnormalities, which implied that corneal epithelial maintenance was abnormal. The *Mp*/+ corneal epithelium was thin, loosely packed and contained goblet cells in older mice. Evidence also suggested that the barrier function was compromised. However, there was no major effect on corneal epithelial cell turnover and mosaic patterns of radial stripes indicated that radial cell movement was normal. Limbal blood vessels formed an abnormally wide limbal vasculature ring, K19-positive cells were distributed more widely than normal and K12 was weakly expressed in the peripheral cornea. This raises the possibilities that the limbal-corneal boundary was poorly defined or the limbus was wider than normal. BrdU label-retaining cell numbers and quantitative clonal analysis suggested that limbal epithelial stem cell numbers were not depleted and might be higher than normal. However, as corneal epithelial homeostasis was abnormal, it is possible that *Mp*/+ stem cell function was impaired. It has been shown recently that the *Mp* mutation involves a chromosome 18 inversion that disrupts the *Fbn2* and *Isoc1* genes and produces an abnormal, truncated fibrillin-2^MP^ protein. This abnormal protein accumulates in the endoplasmic reticulum (ER) of cells that normally express *Fbn2* and causes ER stress. It was also shown that *Fbn2* is expressed in the corneal stroma but not the corneal epithelium, suggesting that the presence of truncated fibrillin-2^MP^ protein in the corneal stroma disrupts corneal epithelial homeostasis in *Mp*/+ mice.

## Highlights

• Heterozygous mutant *Mp*/+ mice have small, abnormal eyes.
• The corneal epithelium is thin, loosely packed and has goblet cells.
• Corneal epithelial cell turnover and radial cell movement appear normal.
• The cornea-limbal border is poorly defined and the limbus appears wider than normal.
• Indirect tests suggest stem cells are not depleted and numbers might be increased.

## 1 Introduction

The corneal epithelium forms the outer barrier layer of the cornea and in adult mice it usually comprises 5–6 cell layers (Smith et al., 2002). The main bulk of the cornea is formed by the underlying corneal stroma, which consists of sparsely distributed keratocytes embedded in a collagen and proteoglycan matrix and below this is a single layer of corneal endothelial cells. It is widely accepted that the adult corneal epithelium is maintained by limbal epithelial stem cells (LESCs), located in the limbus at the junction of the peripheral cornea and conjunctiva and this has been supported recently by lineage tracing experiments (Amitai-Lange et al., 2015, Di Girolamo et al., 2015, Dorà et al., 2015). The LESCs produce transient (or transit) amplifying cells (TACs), which move centripetally across the corneal radius to the centre, within the basal corneal epithelial layer. The TACs divide several times in the basal layer and, after a final division, post-mitotic daughter cells move into the suprabasal layer, become differentiated and are finally lost from the surface by desquamation (Beebe and Masters, 1996).

Maintaining a balance of epithelial cell production and loss is critical for normal corneal epithelial homeostasis (Thoft and Friend, 1983). Many mouse mutants, knockouts and conditional gene knockouts affect corneal morphology and disrupt homeostasis but there are few examples where LESC depletion or loss of function has been demonstrated. For example, the corneas of *PAX6*^*+/*^^*−*^ heterozygous individuals with aniridia, show progressive corneal deterioration with encroachment of conjunctival cells, including goblet cells, onto the cornea, and evidence suggests that this is at least partly caused by LESC deficiency (Ahmad, 2012, Nishida et al., 1995, Puangsricharern and Tseng, 1995, Secker and Daniels, 2008). A similar progressive corneal deterioration occurs in heterozygous *Pax6*^+/−^ mice but direct proof of LESC deficiency in these mice remains elusive (Davis et al., 2003, Ramaesh et al., 2003, Ramaesh et al., 2005). Analysis of mosaics showed that radial cell movement was disrupted in *Pax6*^*+/*^^*−*^ mice but not *PAX77*^*Tg/-*^ transgenic mice, which have elevated Pax6 levels. However, quantitative analysis of stripe numbers in mosaics suggested that LESC clone numbers were reduced in both *Pax6*^*+/*^^*−*^ and *PAX77*^*Tg/-*^ mice (Collinson et al., 2004, Douvaras et al., 2013, Mort et al., 2011).

There is evidence that LESCs are affected in several other genetic mouse models where corneal epithelial homeostasis is impaired, including *Lrig1*^*−/−*^, an *Lhx2* conditional knockout *(K14-Cre;Lhx2*^*fl/fl*^) and *Dstn*^*corn1/corn1*^. Nakamura et al. (2014) showed that human *LRIG1* gene expression was upregulated in holoclone-type corneal cultures (putative stem cells). They then showed that corneal homeostasis was impaired in *Lrig1*^*−/−*^ knockout mice and, after multiple debridements, wound healing was delayed and incomplete. As LESCs are induced to proliferate in order to repair large wounds (Lehrer et al., 1998), the poor wound healing response suggests LESCs are deficient in *Lrig1*^*−/−*^ mice. Similar evidence suggests that the abnormal corneal epithelial morphology, implying impaired corneal homeostasis, which was seen in some *K14-Cre*;*Lhx2*^*fl/fl*^ mice also involved a LESC deficiency (Sartaj et al., 2016). After successive corneal epithelial debridements, wound healing was incomplete in *K14-Cre*;*Lhx2*^*fl/fl*^ mice and their corneal epithelium contained goblet cells and K15-positive cells.

In adult *Dstn*^*corn1/corn1*^ mice, corneal epithelial cells proliferated but did not move radially and the corneal epithelium included goblet cells, K8-positive, conjunctiva-like epithelial cells and regions of hypoplasia (Zhang et al., 2008). Furthermore, BrdU label-retaining cells were present in the cornea as well as the limbus and the authors proposed that the *Dstn*^*corn1/corn1*^ corneal epithelium was not maintained by LESCs in the limbus but by stem cells, within the corneal epithelium. As far as we are aware, the *Dstn*^*corn1/corn1*^ mouse is the only example where differences in label-retaining cell distributions have provided evidence of altered LESC function. This approach was also used to try to determine whether the number of LESCs, identified as label-retaining cells, was depleted in *Pax6*^*+/*^^*−*^ mice but the results were confused by other abnormalities (Douvaras et al., 2013).

Other genetic models of LESC deficiency are required and, in the present study, we have investigated whether *Mp*/+ mice have abnormal corneal homeostasis and LESC deficiency. *Mp*/+ mice are heterozygous for the irradiation-induced *Mp* (micropinna microphthalmia) mutation (Phipps, 1964, Phipps, 1965). Both *Mp*/+ and *Mp/Mp* mice have small ears and small eyes but *Mp/Mp* are more severely affected and usually die at around weaning age. At the time of our investigations, the nature of the *Mp* mutation was not understood but this has now been characterised (Rainger et al., 2013).

Rainger et al. (2013) showed that the *Mp* mutation involves a 660 kb inversion on chromosome 18 that disrupts the *Fbn2* (fibrillin-2) and *Isoc1* (isochorismatase domain containing-1) genes. The Mp inversion, produces an abnormal, truncated fibrillin-2^Mp^ (Fbn2^Mp^) protein and this is thought to cause the abnormal *Mp/Mp* and *Mp*/+ ocular phenotypes, which are more severe than either *Fbn2*^*−/−*^ homozygotes or *Fbn2*^*+/*^^*−*^ heterozygotes, for which no ocular defects have been reported (Shi et al., 2013b). Rainger et al. (2013) also demonstrated that some *Mp/Mp* tissues, including the developing corneal stroma, showed the hallmarks of ER stress. Cells contained intracellular inclusions, suggesting that Fbn2^Mp^ protein accumulated in the endoplasmic reticulum (ER), reduced the secretion of other proteins and perturbed ER homeostasis. This would lead to ER stress and trigger the unfolded protein response, which can also cause cell death. The authors, therefore, proposed that this mechanism explained the worse-than-null phenotypes of both *Mp/Mp* and *Mp*/+ mice (Rainger et al., 2013).

The aims of the present study were to determine whether corneal epithelial morphology and maintenance were abnormal in adult *Mp*/+ mice and if there was evidence of impaired LESC function. We characterised *Mp*/+ corneal epithelial morphology, expression of specific markers and indirectly evaluated permeability. We also investigated whether there was evidence for altered rates of corneal epithelial cell turnover and we compared LESC function indirectly in *Mp*/+ and WT mice by analysing X-inactivation mosaic stripe patterns and BrdU label-retaining cell numbers.

## 2 Materials and methods

### 2.1 Mice

Animal work was approved by the University of Edinburgh Ethical Review Committee and performed in accordance with UK Home Office regulations under project license number PPL 60/3635. Mice of both sexes were used except for the analysis of X-inactivation mosaics when only *XLacZ*^*Tg/-*^ mosaic females were used. The *Mp* mutation (Phipps, 1964, Phipps, 1965) has now been identified as an inversion in chromosome 18 (Rainger et al., 2013) and is designated In(18Fbn2-Isoc1)Mp or In(18)Mp. For simplicity, we have used *Mp*/+, rather than *Mp/Mp*^*+*^, to denote the heterozygote. Heterozygous *Mp*/+ mice, on a mixed genetic background, were provided by Prof. Ian Jackson and Dr. Joseph Rainger (University of Edinburgh) and crossed to the inbred CBA/Ca strain. *Mp*/+ heterozygotes were distinguished from WT littermates by their small ears and eyes (illustrated in Rainger et al., 2013). Heterozygous *Pax6*^*+/Sey-Neu*^ (*Pax6*^*+/*^^*−*^) mice were maintained on a CBA/Ca genetic background and distinguished from *Pax6*^*+/+*^, wild-type (WT) littermates by PCR and eye size (Quinn et al., 1996). H253 strain mice (Tan et al., 1993), ubiquitously expressing the *Tg(Hmgcr-lacZ)H253Sest*, X-linked *nLacZ* transgene (abbreviated to *XLacZ*), were maintained on a mixed C57BL/6 and CBA/Ca genetic background. Hemizygous males, hemizygous females and homozygous females are designated respectively *XLacZ*^*Tg/Y*^, *XLacZ*^*Tg/-*^ and *XLacZ*^*Tg/Tg*^. Both *Mp*/+, *XLacZ*^*Tg/-*^ and WT, *XLacZ*^*Tg/-*^ X-inactivation mosaics were produced by *Mp*/+ female × *XLacZ*^*Tg/Y*^ male crosses. *Pax6*^*+/*^^*−*^, *XLacZ*^*Tg/-*^ mosaics were produced by equivalent crosses between *Pax6*^*+/*^^*−*^ females and *XLacZ*^*Tg/Y*^ males.

### 2.2 BrdU treatment

For acute labelling with BrdU (5-bromo-2’-deoxyuridine; Sigma-Aldrich), 15-week old mice were given single intraperitoneal (i.p.) injections of BrdU (10 mg BrdU/ml in normal saline; 0.2 ml/mouse) at 10:00 a.m. and killed by cervical dislocation following inhalation of gaseous anaesthetic 4, 28 or 52 h later (at 2:00 p.m.). Eyes were removed, fixed and processed for immunohistochemistry as described below. For BrdU pulse-chase identification of label-retaining cells (LRCs), Alzet mini-osmotic pumps (model 1007D; Palo Alto, CA, USA; from Charles River UK Ltd), containing 0.1 ml BrdU solution (50 mg BrdU/ml in normal saline) were surgically implanted under general anaesthetic at 15 or 30 weeks of age. BrdU was delivered continuously (0.5 μl/h) and the pumps were surgically removed after 7 days. Mice were killed 10 weeks later (i.e. when mice were 26 or 41 weeks old) and corneal buttons were processed for immunofluorescence as described below.

### 2.3 Histology and immunohistochemistry on sections

Eyes were fixed in 4% paraformaldehyde (PFA) in phosphate buffered saline (PBS) and some lenses were removed via a posterior incision to facilitate sectioning. Eyes were dehydrated in ethanol, embedded in paraffin wax and 7 μm longitudinal sections were cut and mounted on Polysine slides (VWR, Lutterworth, UK). The cut surface of the wax block was kept wet during sectioning of eyes with lenses to help preserve the lens morphology. Slides were de-waxed in Histo-Clear (from National Diagnostics via Fisher Scientific, Loughborough, UK), rehydrated to 70% ethanol, washed in PBS, stained in haematoxylin, haematoxylin and eosin (H & E) or neutral red and eosin and mounted in DPX (distyrene plasticiser xylene; from BDH via VWR) under coverslips. For periodic acid-Schiff (PAS) staining, de-waxed sections were treated with 0.5% periodic acid (Sigma-Aldrich) for 15 min, washed in water, stained with Schiff’s reagent (Merck Millipore) for 5 min and washed in lukewarm water for 5 min. Slides were then counterstained with haematoxylin, dehydrated, cleared in xylene and mounted on glass slides with DPX and coverslips.

For BrdU and keratin 12 (K12) immunohistochemistry, sections were dewaxed, rehydrated and incubated with 3% hydrogen peroxide for 20 min to quench endogenous peroxidase activity (Ramaesh et al., 2005, Shi et al., 1991). To avoid loss of sections during antigen retrieval, by heating in a microwave (Shi et al., 1991), slides were immersed in citrate buffer (10 mM sodium citrate pH 6.0) in a covered Coplin jar in a 95 °C water bath for 35 min and then the Coplin jar was removed and allowed to cool for 20 min. Slides were washed (3 × 5 min) in PBS-T (0.02% Tween 20 in PBS), treated with blocking serum (10% normal serum, from the species used to raise the secondary antibody, and 1% bovine serum albumin, BSA, in PBS-T) for 1 h at room temperature to block non-specific antibody binding. Slides were next incubated with primary antibody overnight at 4 °C in a humidified chamber, washed (PBS-T; 3 × 5 min), treated with blocking serum (10 min) and incubated with biotinylated secondary antibody (45 min). Slides were then washed (PBS-T; 3 × 5 min) and incubated with avidin-biotin horseradish peroxidase (ABC complex/HRP kit; Dako K0355) for 1 h, washed (PBS-T; 3 × 5 min) and stained with 3-3’diaminobenzidine (DAB isopac, Sigma D9015) for 4–10 min. Finally, sections were counterstained with haematoxylin and mounted in DPX, under coverslips. Negative control slides were incubated with normal blocking serum without the primary antibody, but otherwise treated in the same way.

Two protocols were used for BrdU immunohistochemistry on sections. For BrdU protocol 1 (Ramaesh et al., 2005), the primary antibody was mouse monoclonal anti-BrdU antibody (Becton Dickinson 347580), diluted 1:150 in blocking serum (10% normal goat serum and 1% BSA, in PBS-T). The secondary antibody was biotinylated goat anti-mouse IgG (Dako E0433), diluted 1:200 in blocking serum. For BrdU protocol 2, the primary antibody was sheep anti-BrdU antibody (Fitzgerald Labs, 20-BS17), diluted 1:1000 in blocking serum (10% rabbit serum and 1% BSA in PBS-T). The secondary antibody was biotinylated rabbit anti-sheep (Vector BA 6000), diluted 1:500 in blocking serum. For K12 immunohistochemistry, the primary antibody was Cytokeratin 12 (Santa Cruz Biotechnology sc-17101), diluted 1:500 in blocking serum (10% normal rabbit serum and 1% BSA in PBS-T). The secondary antibody was biotinylated rabbit anti-goat IgG (Vector Labs BA-5000), diluted 1:200 in blocking serum.

### 2.4 Corneal measurements and cell counts on sections

Mid-sections of eyes (identified by counting serial sections) were used for all the measurements and counts. Sections were photographed using a calibrated Zeiss Axiovision 4.8 digital camera system on a Zeiss Axioplan 2 microscope. The Zeiss measuring tool was used to measure the thickness of the whole cornea, corneal stroma (plus endothelium) and corneal epithelium at peripheral, intermediate and central corneal regions (two locations for each region) in sections close to the mid-section in one eye per mouse. The number of corneal epithelial cell layers was also counted. For estimates of basal cell packing density, the numbers of nuclei were counted in measured lengths of basal corneal epithelium in digital photographs using Adobe Photoshop CS6 and the results were expressed as the mean epithelial length per basal cell. All measurements and counts were made by a single investigator (NJD).

To analyse cell proliferation and loss in BrdU immunostained sections, the junction of the cornea and limbus was identified near the ciliary body (or anterior to the irido-corneal angle in *Mp*/+ mice without ciliary bodies). Each basal corneal epithelial cell was scored as positive or negative across the entire corneal diameter, which was retrospectively divided into two peripheral regions (P), two intermediate regions (I) and two central regions (C). The basal labelling index (LI) was calculated as the percentage BrdU positive cells in the basal layer for the whole diameter and separate P, I and C regions. The percentage BrdU-positive cells in the combined suprabasal layers (suprabasal LI) was scored in an equivalent way. To compensate for the thinner corneal epithelium in *Mp*/+ mice, an adjusted suprabasal LI was also calculated, by expressing the number of suprabasal BrdU-positive cells as a percentage of the total cell number in the underlying basal layer rather than the total suprabasal cell number. All counts were made by a single investigator (EJL).

### 2.5 Whole-mount cornea immunofluorescence and confocal imaging

Whole-mount corneal immunofluorescence was based on the method described by Pal-Ghosh et al. (2004), as reported previously (Douvaras et al., 2013). Briefly, eyes were fixed in 4 methanol: 1 DMSO, corneal buttons were dissected, and incubated sequentially (with intermediate PBS-T washes) in blocking serum (1% BSA and 1% goat serum in PBS-T), primary antibody (overnight at 4 °C), blocking serum and secondary antibody (overnight at 4 °C). Primary antibodies used were rat anti-BrdU (Abcam), rabbit anti-K19 (LifeSpan Biosciences) and rat anti-CD31 (BD Pharmingen). Secondary antibodies were goat Alexa 488 Anti-Rat IgG (Invitrogen), goat Alexa 488 Anti-Rabbit IgG (Invitrogen) and goat Alexa 568 Anti-Rat IgG (Invitrogen). After incubating with the secondary antibody, corneas were washed (PBS-T; 4 h), counterstained with the fluorescent nuclear stain TO-PRO3 iodide (Invitrogen) containing RNaseA, flattened with radial cuts and mounted on slides with MoWiol 4-88 (Calbiochem) containing 2.5% DABCO (1,4-diazobicyclooctane). For flat-mounting, 8 radial cuts were made in the WT corneas to create 8 corneal sectors but only 4 radial cuts were made in the smaller *Mp*/+ eyes. Whole-mount corneas, stained with a fluorescently conjugated antibody, were imaged by acquiring overlapping Z-stacks with a Leica TCS NT confocal microscope and exported to ImageJ (http://rsb.info.nih.gov/ij/). The MosaicJ plug-in (http://bigwww.epfl.ch/thevenaz/mosaicj) was used to construct composite images.

Confocal Z-stack images of whole-mount BrdU immunofluorescence were obtained for each sector and BrdU label-retaining cells were identified as BrdU-positive nuclei in maximum projection images of the Z-stacks. ImageJ was used to superimpose a calibrated 340 × 200 μm sampling box on the image across the width of the limbal region in each sector. The number of BrdU-positive nuclei within each sampling box was counted and the mean number of LRCs per sampling box (one sampling box per sector) was calculated for each cornea. To correct for differences in corneal diameters, the mean number of LRCs per sampling box was multiplied by the corneal circumference (calculated from the mean of 3 diameters) and divided by the length of the sampling box (340 μm) to provide an estimate of the number of LRCs in a 200 μm wide ring around the whole limbal circumference. All counts of LRCs were made by a single investigator (PD).

### 2.6 Analysis of X-inactivation mosaic corneas

Dissected whole eyes from *XLacZ*^*Tg/-*^, X-inactivation mosaic mice were lightly fixed in gluteraldehyde, stained with X-gal, post-fixed in 4% paraformaldehyde then either viewed as whole-mounts or embedded in wax, sectioned, counterstained with neutral red and eosin and mounted in DPX under coverslips (Collinson et al., 2002, Douvaras et al., 2013).

To compare growth and cell mixing in corneal epithelia from 3 to 10 week old *Mp*/+, *XLacZ*^*Tg/-*^ and WT, *XLacZ*^*Tg/-*^ mice, mosaic patterns were analysed in histological sections of eyes that had been stained with X-gal. Cells in the basal corneal epithelial layer of the mid-section were counted and scored as β-gal-positive or β-gal-negative across the diameter of the cornea by light microscopy and the numbers of cells in alternate β-gal-positive and β-gal-negative patches were recorded. The number of patches, mean patch length and median patch length were recorded separately for the β-gal-positive and β-gal-negative cell populations and the percentage β-gal-positive cells was calculated. The total numbers of basal cells and patches across the corneal diameter were also recorded. All measurements were made by a single investigator (PD). To compare patch sizes and evaluate cell mixing, the mean patch length was corrected for effects of different proportions of β-gal-positive cells by dividing it by the function 1/(1-p), where p is the proportion of β-gal-positive cells (Hodson et al., 2011, Roach, 1968, West, 1976). Both this corrected mean β-gal-positive patch length and the uncorrected median patch length for the minority cell type (Schmidt et al., 1986) were calculated as described previously (Douvaras et al., 2013).

Radial stripe numbers in whole-mount eyes, dissected at 15 or 30 weeks, were analysed semi-automatically from digital photographs of the cornea, using the ImageJ plugin ’Clonal Tools’ in batch mode as described elsewhere (Mort, 2009, Mort et al., 2011). This provides a ‘corrected stripe number’, which corrects for the probability that stripes would contain multiple adjacent β-gal-positive corneal epithelial clones as described previously (Collinson et al., 2002, Mort et al., 2009). Where correction for differences in eye sizes was required, the corrected stripe number was divided by the corneal circumference. All measurements were made by a single investigator (RLM).

### 2.7 Statistical analysis

GraphPad Prism version 5.0c (GraphPad Software Inc., La Jolla, CA) was used for statistical tests. More than two groups were compared by 1-way analysis of variance (ANOVA), followed by Tukey’s post tests or 2-way ANOVA, followed by Bonferroni’s post tests. Data that were clearly not normally distributed were analysed by Kruskal-Wallis followed by Dunn’s post tests. Two groups were compared by Student’s *t*-test, Welch’s *t*-test, corrected for unequal variances, or Mann-Whitney U-tests.

## 3 Results

### 3.1 Abnormalities in *Mp*/+ eyes

**Fig. 1:**
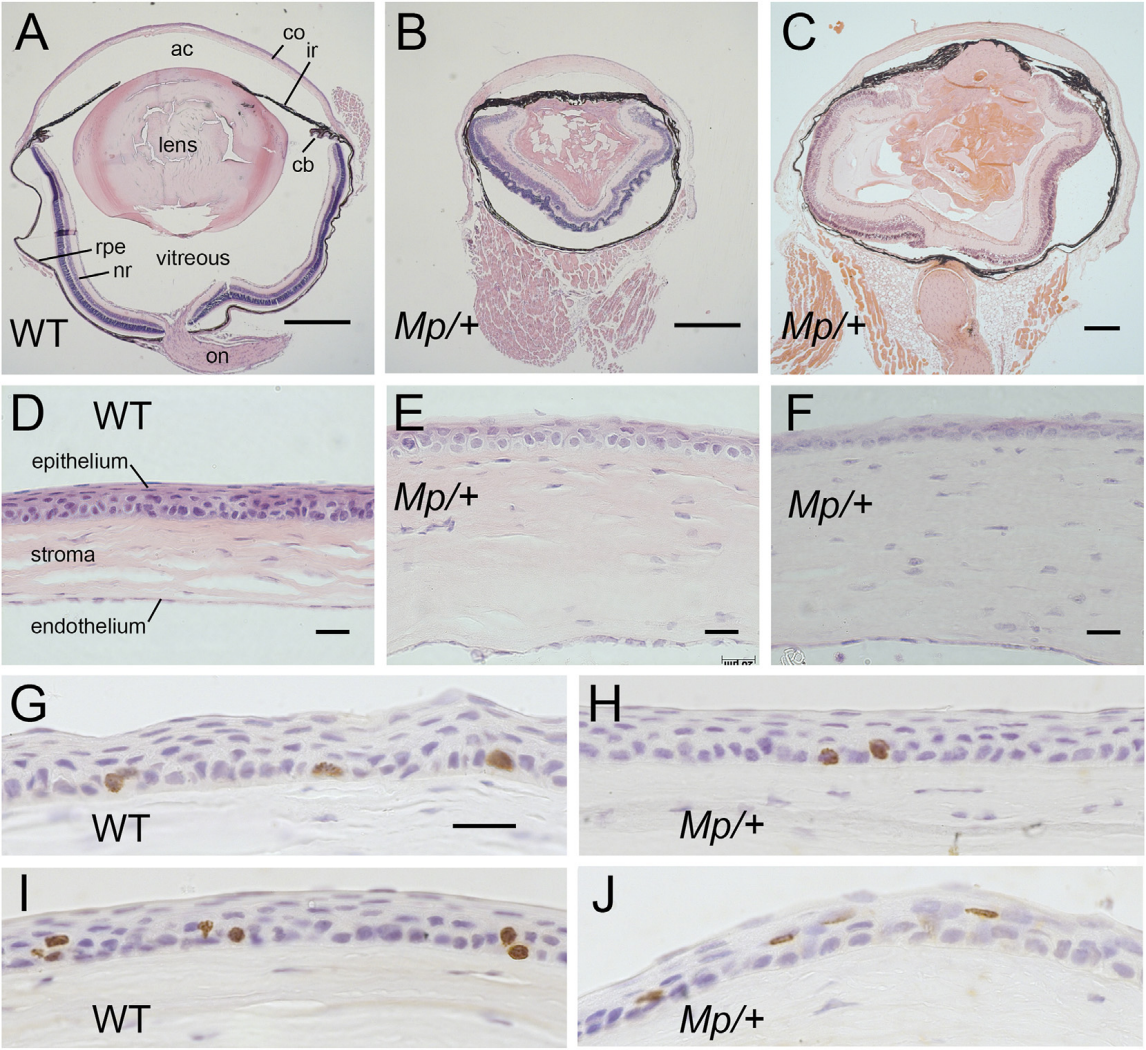
***Mp*/+ eye morphology and BrdU immunohistochemistry (A**–**F)** Haematoxylin and eosin stained sections of wild-type, WT (A) and *Mp*/+ (B, C) eyes plus WT (D) and *Mp*/+ (E, F) corneas. Separation of the retinal pigment epithelium from the neural retina and some lens damage in A are processing artefacts and haematoxylin staining is weak in C. The corneal thickness in E and F may be misleading because A-F are not all mid-sections. Mice were 9 weeks (A, B, D, E), 15 weeks (C) and 1 year (F) old. B and C are representative of *Mp*/+ eyes from eyes that were sectioned from mice at 5 different ages up to one year. **(G**–**J)** Immunohistochemical staining for BrdU in the corneal epithelium (protocol 2 with sheep anti-BrdU and brown DAB endpoint) in WT (G, I) and *Mp*/+ (H, J) eyes. Mice were injected with BrdU at 15 weeks and left for different chase times. Results are illustrated for two chase times. After 4 h the labelling was confined to the basal cell layer (G, H) and after 52 h labelled cells were present in both basal and suprabasal layers (I, J). The photographs are representative of eyes from six 15-week old mice of each genotype at each chase time. (In greyscale photographs the brown DAB nuclear stain appears dark.) Scale bars: A, B = 500 μm; C = 200 μm; D-F = 20 μm. G (for G-J) = 20 μm. Abbreviations: ac, anterior chamber; cb, ciliary body; co, cornea; ir, iris; nr, neural retina, on, optic nerve; rpe, retinal pigment epithelium (with adjacent pigmented choroid); vitreous, vitreous chamber. (For interpretation of the references to colour in this figure legend, the reader is referred to the web version of this article.)

Rainger et al. (2013) showed that at postnatal day (P) 21 *Mp*/+ mice, on a C57BL/6 genetic background had no ciliary body, no vitreous chamber, the anterior chamber was greatly reduced, the lens had cataracts, the retina was folded and contained rosettes but iris development appeared relatively normal. We confirmed that eyes of our adult *Mp*/+ mice on a CBA/Ca genetic background were small, had retinal abnormalities and no ciliary body and that the vitreous chamber was either absent or very small. However, the lens and iris were more significantly affected than previously described (Fig. 1A-C) and this could be at least partly a consequence of the different genetic backgrounds. The iris extended completely across the anterior of the eye without a pupillary opening but did not adhere to the corneal endothelium and the lens was disorganised and sometimes vacuolated. One eye was examined from each of two mice at five different ages (4 weeks–12 months) but no consistent relationship between age and severity of the eye abnormalities was noted (data not shown).

### 3.2 Corneal abnormalities in *Mp*/+ eyes

**Fig. 2:**
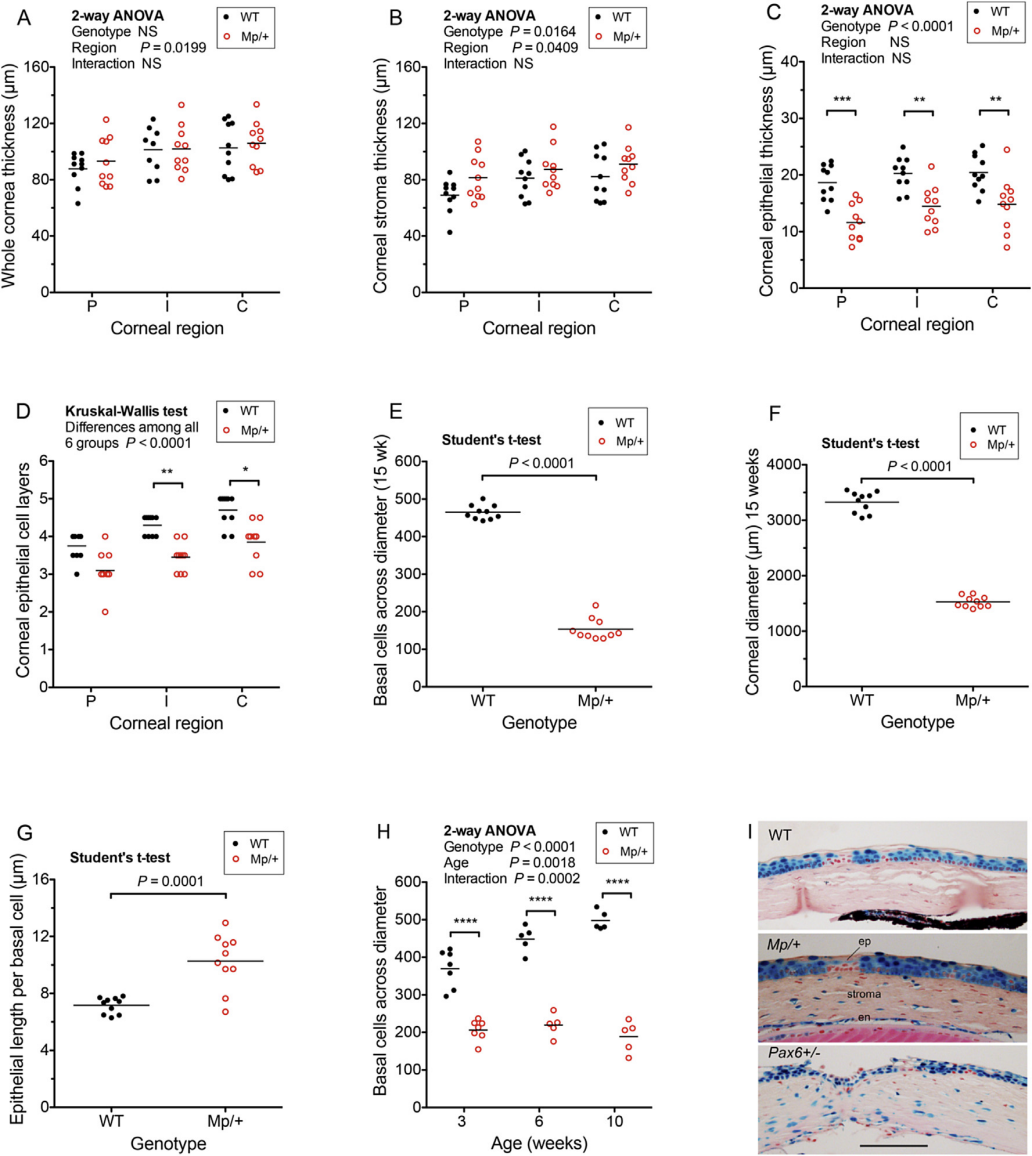
**Corneal measurements and epithelial permeability. (A**–**D)** Comparisons of WT and *Mp*/+ corneal thickness at peripheral (P), intermediate (I) and central (C) corneal regions, shown as whole corneal thickness (A), corneal stroma (plus endothelium) thickness (B), corneal epithelial thickness (C) and number of corneal epithelial cell layers (D). Measurements were made on mid-sections of corneas from right eyes of ten 15-week old mice of each genotype. In A-D, significant genotype differences are shown as asterisks (Bonferroni’s multiple comparison test after 2-way ANOVA for A-C and Dunn’s multiple comparison test after Kruskal-Wallis test for D). **(E**–**G)** Comparisons of WT and *Mp*/+ corneas from 15-week old mice, shown as the number of basal corneal epithelial cells across the corneal diameter (E), the corneal diameter (μm) (F) and the length of corneal epithelium per basal cell (G). For A-G, measurements were made on mid-sections of corneas from right eyes of ten 15-week old mice of each genotype. Significant genotype differences are shown. (**H**) Comparisons of the number of basal corneal epithelial cells across the corneal diameter of WT *XLacZ*^*Tg/-*^ and *Mp*/+ *XLacZ*^*Tg/-*^, X-inactivation mosaics at 3, 6 and 10 weeks. Measurements were made on mid-sections of one eye per mouse from 7 WT and 7 *Mp*/+ mice at 3 weeks and 5 of each genotype at 6 and 10 weeks. Genotype differences at each age were all highly significant by Bonferroni post tests. Each point in the graphs represents one eye and the mean is shown by a horizontal bar. **(I)** Representative sections of corneas from 3-week old *XLacZ*^*Tg/-*^ female X-inactivation mosaic mice that were stained for β-galactosidase (β-gal; blue) and counterstained with eosin and neutral red (pink). β-gal staining is present in the corneal epithelium but not the stroma of WT *XLacZ*^*Tg/-*^ corneas (top photograph) but present in both the corneal epithelium and corneal stroma of *Mp*/+ *XLacZ*^*Tg/-*^ and *Pax6*^*+/*^^*−*^*XLacZ*^*Tg/-*^ corneas. (Eyes from 7 WT, 7 *Mp*/+ and 6 *Pax6*^*+/*^^*−*^ mice were stained). *P < 0.05; **P < 0.01; ***P < 0.001; ****P < 0.0001. Abbreviations: en, endothelium; ep, epithelium; NS, not significant; wk, week. Scale bar in I = 100 μm. (For interpretation of the references to colour in this figure legend, the reader is referred to the web version of this article.)

*Mp*/+ corneas tended to have slightly thinner epithelia than wild-type (Fig. 1D–J) but the thickness of the *Mp*/+ corneal stromas shown in Fig. 1E, F may be misleading because not all sections illustrated in Fig. 1A–F were mid-sections. Mid-sections of 10 WT and 10 *Mp*/+ corneas, from the series illustrated in Fig. 1G–J, were measured. Both the WT and *Mp*/+ corneas were thicker in the central cornea than at the periphery (Fig. 2A), so the genotypes were compared in three regions: peripheral (P), intermediate (I) and central (C) cornea. Although the overall corneal thickness did not differ significantly between genotypes, the stroma (plus endothelium) was slightly thicker in *Mp*/+ than WT eyes (Fig. 2A, B). This difference between genotypes reached significance by 2-way ANOVA for the whole cornea but not for individual regions. Conversely, *Mp*/+ corneal epithelia were significantly thinner than WT in all regions and had significantly fewer cell layers in intermediate and central corneal regions (Fig. 2C, D). As expected for the small eye size, the corneal diameter and the number of cells across the corneal diameter were significantly reduced in *Mp*/+ mice by 15 weeks (Fig. 2E, F). The length of corneal epithelium per basal cell (measured diameter divided by the number of basal cell nuclei across the diameter) was also significantly greater in *Mp*/+ compared to WT corneas (Fig. 2G), indicating that *Mp*/+ cells were larger and/or more loosely packed at 15 weeks.

Histological sections cut after staining 3-week old, WT *XLacZ*^*Tg/-*^ mosaic mouse eyes for β-gal showed that staining was restricted to the corneal epithelium, whereas β-gal-positive nuclei were also present in the corneal stroma and endothelium of *Mp*/+, *XLacZ*^*Tg/-*^ mosaics (Fig. 2I). As intact eyes were stained before sections were cut, this difference suggests that the thinner, more loosely-packed *Mp*/+ corneal epithelium is more permeable to liquids than normal, so stain was able to penetrate to the stroma and endothelium. β-gal staining of the corneal stroma and endothelium was also observed in the *Pax6*^*+/*^^*−*^ *XLacZ*^*Tg/*^^*−*^ (Fig. 2I), which has a thin and fragile corneal epithelium (Ramaesh et al., 2003).

Sections of WT, *XLacZ*^*Tg/-*^ and *Mp*/+, *XLacZ*^*Tg/-*^ mosaics eyes from 3-, 6- and 10-week old mosaic mice were also used to compare corneal growth in WT and *Mp*/+ eyes. *Mp*/+ corneas had fewer basal epithelial cells across the corneal diameter than WT at each age (Fig. 2H). Separate one-way ANOVAs for each genotype showed significant differences in cell counts across the diameter among ages for WT (*P* = 0.0003) but not *Mp*/+ eyes (*P* = 0.3693), indicating that *Mp*/+ corneas were smaller in diameter by 3 weeks and failed to grow normally between 3 and 10 weeks.

### 3.3 Corneal epithelial cell turnover

**Fig. 3:**
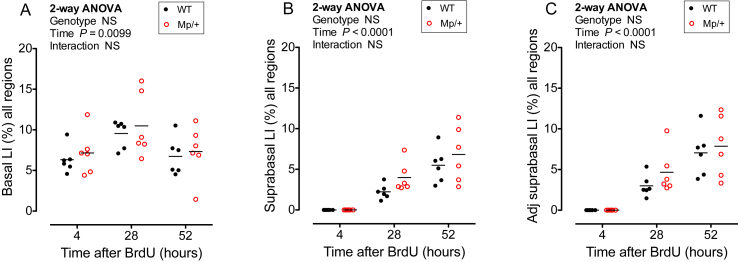
**Corneal epithelial turnover**. WT and *Mp*/+ mice were injected with BrdU at 15 weeks and the percentage BrdU-positive cells (labelling index, LI) in different layers of the corneal epithelium (excluding the limbus) was determined by immunohistochemistry of mid-sections for chase periods of 4, 28 and 52 h. (**A**) Basal BrdU LI (BrdU positive basal cells as a percentage of total basal cells). (**B**) Suprabasal BrdU LI (BrdU positive suprabasal cells as a percentage of total suprabasal cells). (**C**) Adjusted (Adj) suprabasal BrdU LI (BrdU positive suprabasal cells as a percentage of total basal cells to allow for any differences in number of cell layers). Results for 2-way analyses of variance (ANOVAs) for the BrdU LI across the whole corneal diameter are shown. There were no significant differences between WT and *Mp*/+ genotypes. The right eyes were scored for six mice of each genotype at each of the three time points. Mice of both sexes were used. Each point in the graphs represents one eye and the mean is shown by a horizontal bar. NS, not significant. Results are presented separately for the peripheral, intermediate and central corneal regions in Supplementary Fig. S1.

The thinner, more loosely packed corneal epithelium of *Mp*/+ mice could indicate that the rate of corneal epithelial cell turnover is abnormal so we examined incorporation of BrdU by immunohistochemistry. Preliminary immunohistochemistry with a mouse monoclonal anti-BrdU and a biotinylated goat anti-mouse IgG secondary antibody (protocol 1) worked well with WT eyes, as reported previously (Ramaesh et al., 2005), but produced heavy background staining with *Mp*/+ eyes, even without primary antibody or if *Mp*/+ mice were not injected with BrdU (not shown). However, BrdU labelled cells were identified successfully in both WT and *Mp*/+ corneas by immunohistochemistry using a sheep anti-BrdU antibody (protocol 2), as shown in Fig. 1G-J, and were counted (Fig. 3).

Four hours after labelling, BrdU-positive cells were confined to the basal layer. The basal layer labelling index (LI) increased between 4 and 28 h in both *Mp*/+ and WT corneas and then declined by 52 h (Fig. 3A). There were no significant differences between the genotypes whether the basal layer LIs were calculated for the whole cornea (Fig. 3A) or separately for peripheral, intermediate and central regions (Supplementary Fig. S1A-C). At four hours, the basal layer LIs for the whole cornea were very similar for the two genotypes: 6.32 ± 1.32% (mean ± 95% confidence interval) for WT and 7.15 ± 2.12% for *Mp*/+. This implies that there was no major effect on the rate of cell proliferation.

Only basal cells divide and incorporate BrdU so suprabasal cells only become labelled when basal cells move to the suprabasal layers. This is irreversible and is the first step towards cell loss, so more rapid accumulation of BrdU-positive cells in the suprabasal layers in one group would suggest a higher rate of cell loss, unless the total number of corneal epithelial cells (and thus the thickness of the epithelium) changed progressively over time. By 28 h BrdU-positive cells appeared in the suprabasal layers of both genotypes. The suprabasal LI was slightly higher in *Mp*/+ than WT eyes at both 28 h and 52 h (Fig. 3B) but the difference was only significant when peripheral, intermediate and central regions were analysed separately for each genotype at 28 h (*P* = 0.0221 for genotype differences by 2-way ANOVA; Supplementary Fig. S1D-F). However, when the LI was adjusted to compensate for the reduced number of *Mp*/+ suprabasal layers there were no significant differences between genotypes, either for the whole cornea (Fig. 3C) or for the three separate regions (Supplementary Fig. S1G–I). At 28 h, the adjusted suprabasal LIs for the whole cornea were 2.99 ± 1.07% for WT and 4.66 ± 2.14% for *Mp*/+. At this time, in WT corneas 23.4 ± 6.4% of BrdU-labelled cells were in the suprabasal layers and for *Mp*/+ corneas this was slightly higher (30.4 ± 6.7%) but this difference was not significant by student’s t-test (*P* = 0.1712). Thus, despite some trends for a small increase in suprabasal labelling, there was no evidence that the *Mp*/+ genotype had a major effect on corneal epithelial cell proliferation or loss. Although, this does not exclude the possibility that cell turnover is increased marginally in *Mp*/+ corneas, a larger experiment would be required to investigate this possibility.

### 3.4 Evidence for normal radial corneal epithelial cell movement in adult *Mp*/+ mice

**Fig. 4:**
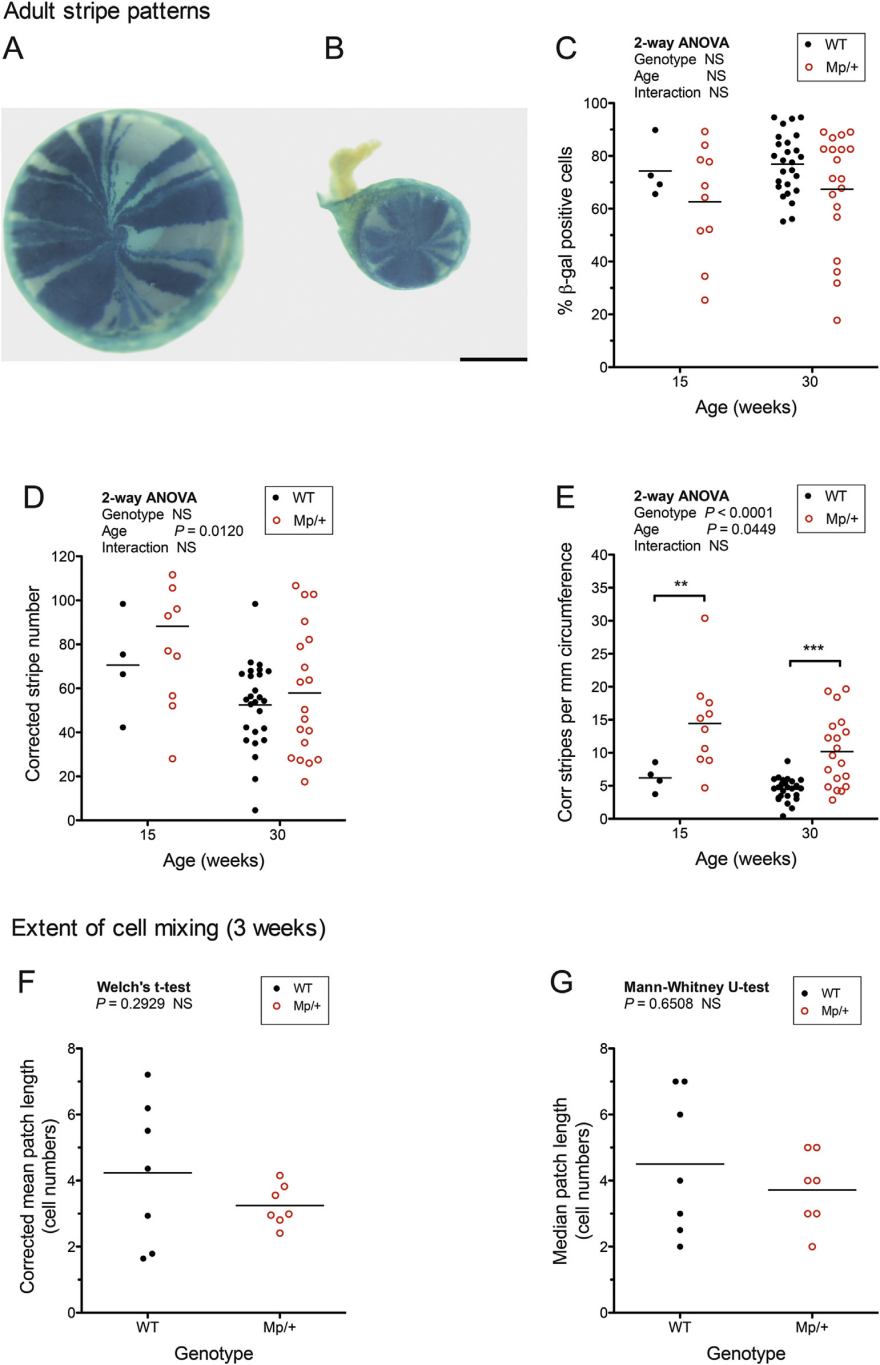
**Mosaic patterns in the corneal epithelium of *Mp*/+ and WT *XLacZ***^***Tg/-***^**X-inactivation mosaics**. (**A, B**) Adult, WT *XLacZ*^*Tg/-*^ (A) and *Mp*/+ *XLacZ*^*Tg/-*^ (B) X-inactivation mosaic eyes stained for β-gal, showing characteristic radial stripes in the corneal epithelium. (**C–E**) Quantitative analyses of adult striped patterns illustrated in A and B for 4 15-week WT, 10 15-week *Mp*/+, 26 30-week WT and 19 30-week *Mp*/+ eyes. (**C**) Percentage of β-gal-positive cells in corneal epithelia from *Mp*/+ and WT X-inactivation mosaics at 15 and 30 weeks. There were no significant differences between genotypes or ages by 2-way ANOVA. (**D**) Corrected stripe numbers per cornea differed significantly for age but not genotype by 2-way ANOVA. Individual pairwise comparisons by Bonferroni multiple comparison tests showed that the corrected stripe numbers per cornea differed significantly between 15 and 30 weeks for *Mp*/+ (*P* < 0.05) but not WT corneas. There were no significant differences between genotypes at either age. (**E**) Corrected (Corr) stripe numbers per mm of circumference in corneal epithelia from *Mp*/+ and WT X-inactivation mosaics at 15 and 30 weeks differed significantly for age and genotype by 2-way ANOVA (shown on graph). For individual pairwise comparisons, Bonferroni multiple comparison tests revealed significant differences between ages in *Mp*/+ mice (*P* < 0.05) and between genotypes at both 15 and 30 weeks (shown on graph). **(F, G)** Analysis of the extent of cell mixing by comparing β-gal-positive patch lengths across the diameter of corneal epithelia in eyes from 7 *Mp*/+ and 7 WT X-inactivation mosaics at 3 weeks (one eye per mouse). Patch lengths (in cell numbers) were compared as the corrected mean patch length (F), as explained in the text, and the uncorrected median patch length of the minor cell population (G) and neither differed significantly between genotypes. Each point in the graphs represents one eye and the mean is shown by a horizontal bar. **P < 0.01; ***P < 0.001. Scale bar (A, B) = 1 mm.

It is widely accepted that corneal epithelial cells move centripetally across the corneal radius from stem cells in the limbus (Buck, 1985, Di Girolamo et al., 2015, Nagasaki and Zhao, 2003). The radial striped patterns in adult *XLacZ*^*Tg/-*^, X-inactivation mosaics reflect this movement of corneal epithelial cells. *Mp*/+, *XLacZ*^*Tg/-*^ mosaics corneas showed similar patterns to WT, *XLacZ*^*Tg/-*^ eyes with radial stripes extending between the limbal region and central cornea (Fig. 4A, B), implying that normal radial movement occurs in *Mp*/+ eyes.

### 3.5 Indirect test for LESC deficiency in adult *Mp*/+ mice from analysis of X-inactivation mosaics

We used two indirect methods to investigate whether the thinner *Mp*/+ corneal epithelium was associated with limbal epithelial stem cell (LESC) deficiency. First, we compared striped patterns quantitatively in *Mp*/+, *XLacZ*^*Tg/-*^ and WT, *XLacZ*^*Tg/-*^ X-inactivation mosaics, as described elsewhere (Collinson et al., 2002, Collinson et al., 2004, Douvaras et al., 2013, Mort et al., 2009, Mort et al., 2011). The observed number of β-gal-positive and β-gal-negative radial stripes was corrected to adjust for the expected frequency of adjacent stripes of the same cell population (β-gal-positive or β-gal-negative), as described in the Materials and Methods. The resultant corrected stripe number is not likely to be numerically equal to the number of LESCs because LESCs are expected to be arranged in small coherent clonal groups. However, the corrected stripe number is an estimate of the number of coherent clones of active LESCs (Collinson et al., 2002, Mort et al., 2009). Differences in corrected stripe numbers between *Mp*/+ and WT eyes are, therefore, expected to be proportional to the differences in numbers of active LESCs, as long as the mean number of LESCs per coherent clone does not differ significantly between genotypes.

The percentage of β-gal-positive cells did not differ significantly between *Mp*/+, *XLacZ*^*Tg/-*^ and WT, *XLacZ*^*Tg/-*^ genotypes for the two ages studied (15 and 30 weeks; Fig. 4C). The corrected stripe numbers and, by implication, the number of coherent clones of LESCs declined with age in both genotypes (Fig. 4D). These age differences were statistically significant for the *Mp*/+ *XLacZ*^*Tg/-*^ mosaics but not the WT, *XLacZ*^*Tg/-*^ controls but this may be because few WT, *XLacZ*^*Tg/-*^ corneas were analysed at 15 weeks.

Despite the much smaller eye size, the corrected stripe number per eye was not lower in *Mp*/+, *XLacZ*^*Tg/-*^ mosaics than WT, *XLacZ*^*Tg/-*^ controls at either 15 or 30 weeks (Fig. 4D). After allowing for the much smaller circumferences of *Mp*/+, corneas, the corrected stripe number per mm of circumference, at the corneal-limbal border, was significantly higher in *Mp*/+ *XLacZ*^*Tg/-*^ mosaics than WT, *XLacZ*^*Tg/-*^ controls at both 15 and 30 weeks (Fig. 4E). This suggests that active coherent clones of LESCs were distributed more densely around the limbal circumference in *Mp*/+ eyes.

However, a higher concentration of LESC clones need not imply a higher concentration of LESCs, if β-gal-positive LESCs were more randomly distributed in *Mp*/+ eyes, so there were fewer LESCs in each coherent clone of LESCs. This could occur, for example, if β-gal-positive and β-gal-negative cells were initially more finely intermixed in the developing *Mp*/+ ocular surface than in WT (Fig. 1 in Douvaras et al., 2013). To test whether cell mixing differed before the LESCs were activated and the stripes emerged (at around 5 weeks; Collinson et al., 2002), the sizes of β-gal-positive patches in the basal layer of the corneal epithelium were compared in 3-week old *Mp*/+, *XLacZ*^*Tg/-*^ and WT, *XLacZ*^*Tg/-*^ X-inactivation mosaics (Fig. 2I), as described in the Materials & Methods. Neither the corrected mean lengths of β-gal-positive patches nor the uncorrected median lengths of the minor cell population in the corneal epithelium differed significantly (Fig. 4F, G). Thus, there was no evidence that cells were more finely mixed in the *Mp*/+ocular surface before stem cells were activated and so no evidence that β-gal-positive LESCs in *Mp*/+ eyes were likely to be arranged in smaller coherent clonal groups than in WT eyes. It, therefore, remains possible that LESCs were distributed more densely around the limbal circumference in adult *Mp*/+ eyes. In any case, the analysis of stripe numbers clearly provides no evidence for a depletion of active LESCs in *Mp*/+ mice.

### 3.6 Indirect test for LESC deficiency from label-retaining cells in adult *Mp*/+ mice

**Fig. 5:**
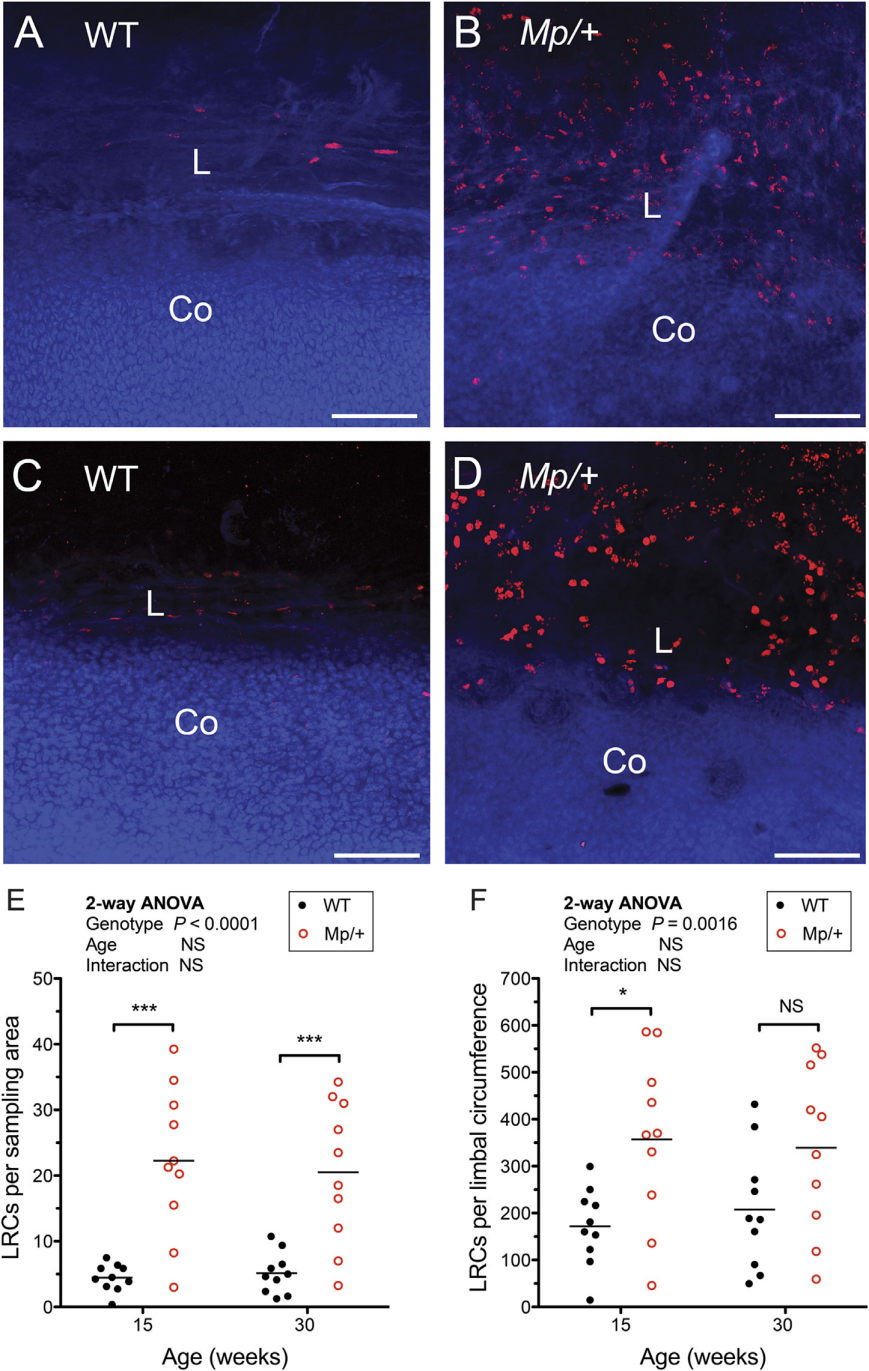
**Identification of label-retaining cells in the limbal region of the *Mp*/+ and wild-type ocular surface epithelium**. **(A**–**D)** Examples of BrdU label-retaining cells in limbal region after 1-week BrdU exposure and 10-week chase period. Representatives of four groups are shown: WT mice labelled at 15 weeks (A), *Mp*/+ mice labelled at 15 weeks (B), WT mice labelled at 30 weeks (C) and *Mp*/+ mice labelled at 30 weeks (D). **(E, F)** Quantitative analysis of label-retaining cells in 10 eyes in each of the four groups shown in A-D. (One eye was analysed per mouse and mice of both sexes were included.) Comparisons are shown as the number of label-retaining cells (LRCs) per 340 × 200 μm sampling area (E) and LRCs per 200 μm wide ring around the limbal circumference (F). In F, the number of LRCs per ring was calculated separately for each eye as LRCs per sampling area × limbal circumference/length of sampling area. In both E and F, two-way ANOVAs showed significant differences for genotypes but not for ages or interactions. Pairwise differences between *Mp*/+ and WT genotypes analysed by Bonferroni multiple comparison tests are shown in the figure. Each point in the graphs represents one eye and the mean is shown by a horizontal bar. *P < 0.05; ***P < 0.001; NS, not significant. Abbreviations: L: Limbus; Co: Cornea. Red immunofluorescence is BrdU and appears light in greyscale photographs; blue is TO-PRO3 iodide counterstain. Scale bars in A-D = 100 μm. (For interpretation of the references to colour in this figure legend, the reader is referred to the web version of this article.)

The second indirect method we used to compare stem cell numbers in WT and *Mp*/+ mice was to compare the numbers of label-retaining cells (LRCs). These were expected to be predominantly putative slow cycling or quiescent stem cells (Cotsarelis et al., 1989, Ksander et al., 2014, Lehrer et al., 1998, Li and Clevers, 2010, Pajoohesh-Ganji et al., 2006, Zhao et al., 2009). We exposed WT and *Mp*/+ mice to BrdU for 7 days at 15 or 30 weeks of age and identified LRCs in the limbal region by whole-mount BrdU immunofluorescence after a 10-week chase period (at 26 or 41 weeks). LRCs were identified by confocal microscopy in each sector of flat mounted cornea buttons (Fig. 5A–D) and counted in a 340 × 200 μm sampling box superimposed over the limbal region. LRCs were restricted to the limbal region in WT eyes and almost exclusively restricted to the limbal region in *Mp*/+ eyes (Fig. 5B, D). The mean number of LRCs per limbal sampling area did not differ significantly between ages for either genotype but it was significantly greater in *Mp*/+ than in WT eyes, for both ages (Fig. 5E). As *Mp*/+ eyes are smaller than WT eyes, we also estimated the number of LRCs within a 200 μm wide ring (the width of the sampling box) around the whole limbal circumference. The mean number of LRCs remained greater in *Mp*/+ eyes when expressed as LRCs per limbal circumference for both age groups but this was only significant for those labelled at 15-weeks (Fig. 5F). The comparison of LRC numbers clearly provided no evidence that *Mp*/+ LESC numbers are depleted.

### 3.7 Distribution of keratin 12-positive and PAS-positive cells in the *Mp*/+ ocular surface

**Fig. 6:**
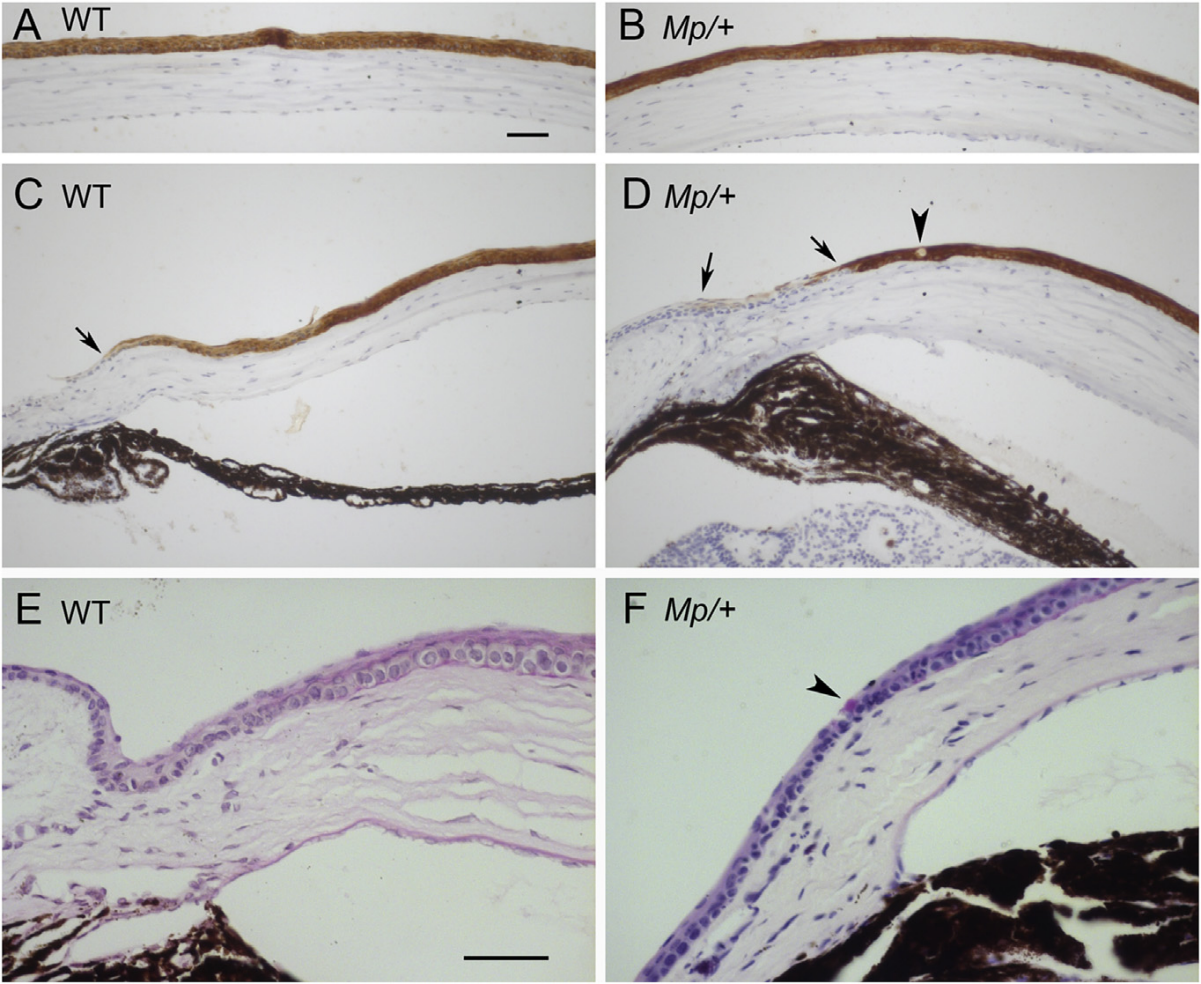
**Immunohistochemistry for K12 and PAS staining of goblet cells. (A**–**D)** Immunohistochemistry for K12 (dark brown DAB endpoint) on sections, showing strong staining in the central corneal epithelium of WT (A) and *Mp*/+ (B) eyes. In the periphery, K12 staining extends to the corneal-limbal boundary, which in the WT eye is approximately level with or slightly anterior to the ciliary body (C). K12 staining also extends to the periphery of the *Mp*/+ corneal epithelium (D) but there is a short region of weak and patchy staining between the two arrows in D. The arrowhead in D indicates a K12-negative putative goblet cell in the peripheral *Mp*/+ corneal epithelium. **(E, F)** Magenta coloured periodic acid-Schiff (PAS) staining for polysaccharides with blue-purple haematoxylin nuclear stain in sections of eyes from 6-month old WT (E) and *Mp*/+ (F) mice (haematoxylin stain is stronger in F than E). The arrowhead in F indicates a PAS-positive goblet cell in the peripheral *Mp*/+ corneal epithelium (not readily distinguishable in greyscale photographs). Scale bars: A (for A-D) and E (for E, F) = 50 μm. (For interpretation of the references to colour in this figure legend, the reader is referred to the web version of this article.)

Histological sections of three WT and three *Mp*/+ eyes from 15-week old mice were immunostained for the corneal epithelial marker keratin 12 (K12) and both genotypes showed strong K12 staining throughout the central corneal epithelium (Fig. 6A, B). K12 staining was present in the peripheral corneal epithelium but not in the limbus of WT eyes and the staining boundary was approximately level with the ciliary body (Fig. 6C). K12 staining was also strong in the peripheral corneal epithelium of *Mp*/+ eyes but there was a short length of weak and patchy staining at the limbal-corneal boundary (Fig. 6D). However, as *Mp*/+ eyes had no ciliary body it was difficult to determine whether the weak staining was in the peripheral cornea or the limbus. Although most *Mp*/+ peripheral corneal epithelial cells were strongly K12-positive, there were some isolated unstained cells, suggesting the presence of goblet cells or other atypical corneal epithelial cells in the *Mp*/+ eye (Fig. 6D).

To determine whether these isolated K12-negative cells in the peripheral corneal epithelium of *Mp*/+ eyes were goblet cells, some corneas were stained for polysaccharides with periodic acid-Schiff (PAS) reagent. Eyes from two mice at each of 5 ages (4 weeks, 9 weeks, 15 weeks, 6 months and 12 months) for each genotype were stained for PAS. No PAS-positive goblet cells were detected in the WT corneal epithelium at any age but some PAS-positive goblet cells were present in the peripheral corneal epithelium in sections of eyes from 3 of the *Mp*/+ mice (both 6-month eyes and one of the two 12-month eyes), as shown in Fig. 6E, F. This suggests that the isolated cells, undetected by K12 immunohistochemistry, were goblet cells.

### 3.8 Distribution of blood vessels and keratin 19-positive cells in the *Mp*/+ ocular surface

**Fig. 7:**
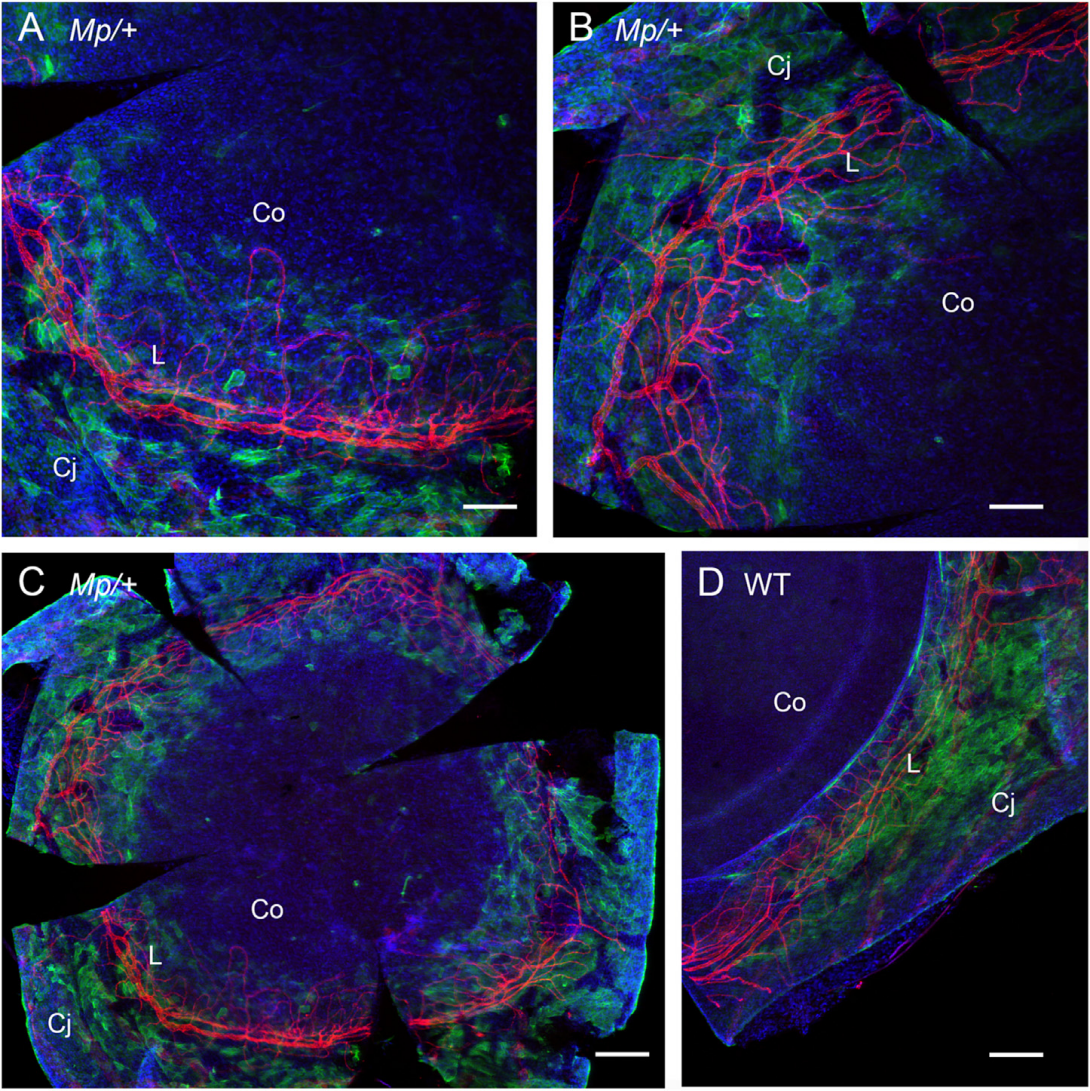
**Immunofluorescence of limbal blood vessels and keratin 19**. Z-stack confocal images (projected to a single image based on maximum intensities) of whole-mount double immunofluorescence for CD31 (red or light vessel outlines in greyscale photographs) in blood vessels and keratin 19, K19 (green or light patchy staining in greyscale photographs) in the ocular surface epithelia of *Mp*/+ (A–C) and WT (D) eyes. Both CD31-positive blood vessels and keratin 19-positive epithelial cells are restricted to the conjunctiva and limbus in WT eyes but in *Mp*/+ eyes the blood vessels loop further from the limbal vasculature ring and there are K19-positive cells in the peripheral cornea. Scale bars: A, B = 100 μm; C, D = 200 μm. Abbreviations: Co, cornea; Cj, conjunctiva; L, limbus. (For interpretation of the references to colour in this figure legend, the reader is referred to the web version of this article.)

We next used confocal microscopy to visualise double immunofluorescence of keratin 19 (K19) and the blood vessel marker, CD31. Blood vessels are usually absent from the cornea and restricted to a characteristic limbal vascular ring in the limbal stroma (Morrison et al., 1995), which helps identify the limbus. K19 is a marker of the limbal epithelium and conjunctiva but is not usually expressed in the mouse corneal epithelium (Yoshida et al., 2006). K19-positive cells in the *Mp*/+ ocular surface (Fig. 7A-C) extended further from the main vessels of the limbal vascular ring than in the WT mice (Fig. 7D) and they appeared to encroach into the peripheral cornea. Furthermore, in *Mp*/+ eyes, some small blood vessels appeared to enter the *Mp*/+ cornea and loop back to the main limbal vascular ring (Fig. 7A-C). This differs from WT, where blood vessels do not extend far towards the cornea from the main limbal vascular ring (Fig. 7D; Fig 5 in Douvaras et al., 2013, Zhao et al., 2009).

## 4 Discussion

### 4.1 How might the *Mp* mutation affect corneal epithelial homeostasis?

It is now known that the *Mp* mutation results in the accumulation of abnormal Fbn2^Mp^ protein, which causes ER stress in *Mp*/+ cell types that normally express *Fbn2* (Rainger et al., 2013). Although *Fbn2* is not expressed in the corneal epithelium it is expressed in some ocular tissues during development of WT embryos (Rainger et al., 2013, Shi et al., 2013a). Rainger et al. also showed that *Fbn2* was expressed in several tissues of fetal *Mp/Mp* eyes, including the corneal mesenchyme, and that protein was retained intracellularly rather than secreted (Rainger et al., 2013). *Mp*/+ cells that express *Fbn2* will produce the abnormal Fbn2^Mp^ protein as well as Fbn2. Thus, proteins will be retained within the cell, causing ER stress and affecting the composition of the extracellular matrix.

Cell types that do not express *Fbn2* may also be affected if they interact with Fbn2^Mp^-positive cells. Although *Fbn2* is not expressed in the corneal epithelium, *Mp*/+ corneal epithelial development and maintenance could be affected indirectly by ER stress in adjacent tissues. The expression of *Fbn2* in the developing corneal mesenchyme and irido-corneal angle (Rainger et al., 2013, Shi et al., 2013a) suggests that ER stress is likely to occur in the corneal stroma, limbal stroma, corneal endothelium, trabecular meshwork and Schlemm’s canal. Thus, ER stress might perturb *Mp*/+ corneal epithelial homeostasis if ER stress in the corneal stroma affects the corneal epithelium or if ER stress in the limbal stroma affects the LESC niche and so compromises LESC function.

### 4.2 Corneal and limbal abnormalities in *Mp*/+ eyes

The main aims were to determine whether corneal epithelial maintenance was abnormal in adult *Mp*/+ mice and if LESC function was compromised. The significant trend for the *Mp*/+ corneal stroma to be thicker than normal could be a direct effect of ER stress within the corneal stroma or endothelium. For example, this might be mediated via poor regulation of fluid retention by the corneal endothelium. Further investigations are required to determine whether ER stress has a direct effect on the *Mp*/+ corneal endothelium or corneal stroma and also identify any quantitative or qualitative changes in keratocytes or stromal lamellae.

The *Mp*/+ corneal epithelium was thinner than normal, cells were loosely packed and results suggested that the barrier function was compromised. The presence of PAS-positive goblet cells in the peripheral corneal epithelium of some older *Mp*/+ mice but not WT mice could reflect incursion of conjunctival cells and LESC deficiency (Nishida et al., 1995), abnormal differentiation (Ramaesh et al., 2003) or possibly a response to wounding (Pajoohesh-Ganji et al., 2012). Regardless of how they arise, the presence of goblet cells is abnormal. Together, the evidence for a thin and loosely packed corneal epithelium, which accumulates goblet cells in older mice, implies that corneal epithelial homeostasis was abnormal.

Investigations with the K12 and K19 markers indicate that the *Mp*/+ ocular surface is abnormal. The *Mp*/+ corneal epithelium had a short length of weak and patchy K12 staining at the limbal-corneal boundary, suggesting differentiation of cells in this region was abnormal. However, it was not clear whether this region was in the peripheral cornea or limbus. Conversely, K19-positive epithelial cells occupied a broader region than normal and this region was defined by an unusually extensive network of capillaries, extending from the *Mp*/+ limbal vascular ring. However, this does not appear to be equivalent to neovascularisation as the blood vessels looped back to the *Mp*/+ limbus, rather than extending towards the centre of the cornea.

The normal striped pattern in adult *Mp*/+ *XLacZ*^*Tg/-*^ mosaics implies that the radial movement of corneal epithelial cells was unaffected in *Mp*/+ eyes, unlike some other genotypes, which either produce abnormal stripes or no stripes in mosaic systems (Collinson et al., 2004, Mort et al., 2012, Zhang et al., 2008). This evidence for normal cell movement in the *Mp*/+ corneal epithelium is also consistent with the notion that the enlarged vasculature in *Mp*/+ eyes differs from neovascularisation. This is because corneal neovascularisation is often associated with abnormal corneal epithelial cell movement (Zhao et al., 2011). For example, both occur in *Dstn*^*corn1/corn1*^ and *Pax6*^+/−^ mice (Collinson et al., 2004, Mort et al., 2011, Ramaesh et al., 2003, Zhang et al., 2008, Zhao et al., 2011).

The distributions of K12, K19 and blood vessels are consistent with the possibility that the *Mp*/+ limbus is wider than normal. If so, the expanded domain of K19-positive cells and larger network of blood vessels would be entirely within an enlarged limbus. Further studies are required to characterise the *Mp*/+ limbus more fully and identify whether, in *Mp*/+ eyes, (i) the blood vessels and K19-positive cells enter the peripheral cornea, (ii) the corneal-limbal boundary is poorly defined, perhaps with an abnormally differentiated zone that includes both K12-positive and K19-positive cells, or (iii) the *Mp*/+ limbus is wider than normal.

### 4.3 Limbal epithelial stem cells in *Mp*/+ mice

Quantitative studies of X-inactivation mosaic mice showed that the mean corrected stripe number per eye was similar in *Mp*/+ and WT mosaic corneas, despite the large difference in eye size and corneal circumference. This implies that *Mp*/+ and WT corneal epithelia are maintained by a similar number of LESC clones, which are distributed more densely around the smaller *Mp*/+ limbal circumference. This might occur, for example, if similar numbers of LESCs are specified in *Mp*/+ and WT eyes early in development (before the corneas diverged in size) but clones of LESCs become more densely distributed around the circumference because the *Mp*/+ cornea stops growing. This could result in either more LESC clones per area of limbus (perhaps with fewer LESCs per clone) or the formation of a wider limbus, as discussed above, with a smaller circumference and a more normal concentration of LESC clones and LESCs per area of limbus. In either case, the number of LESC clones per mm of circumference would be greater than usual in *Mp*/+ eyes. Although adult stripe patterns in X-inactivation mosaics only provide indirect information about active stem cells, it is clear that these results provide no evidence for depletion of active LESCs in *Mp*/+ mice, even without taking into account the smaller *Mp*/+ limbal circumference.

There was a trend for the mean corrected stripe number per cornea and, by implication, the number of coherent clones of LESCs to decline with age in both *Mp*/+ and WT mosaic corneas. This has been shown previously for WT mosaics (Collinson et al., 2002, Mort et al., 2009) and could occur if (i) more LESCs become quiescent during ageing, (ii) some LESCs are lost (so numbers of active LESCs and LESC clones decrease) or (iii) some LESCs are replaced by neighbouring LESCs by stochastic neutral drift (so LESC clone numbers decrease but LESC numbers remain unchanged; Mort et al., 2012).

The label-retaining cell (LRC) method was originally used in the ocular surface to help identify the limbus as the location of putative stem cells (Cotsarelis et al., 1989). Our observation that *Mp*/+ mice had significantly more BrdU LRCs per sampling area than WT mice clearly provides no evidence for depletion of LESCs in *Mp*/+ mice. Although most LRCs are likely to be putative stem cells, it is not a specific method and other slow-cycling cell types might be included as LRCs. For example, when short chase times (4 weeks) were used in rats, some Langerhans cells were included as LRCs (Chen et al., 2007). Also, any labelled cell that stops dividing when it terminally differentiates and is retained in the field that is imaged, would be identified as an LRC. We cannot rule out the possibility that *Mp*/+ LRCs included slow dividing or differentiated cell types that are less numerous among WT LRCs. For example, if proliferating and differentiating vascular cells were present in the enlarged *Mp*/+ limbal vascular ring this might contribute to the increased number of LRCs in the *Mp*/+ limbus but is unlikely to be the full explanation.

Although not all LRCs are LESCs, they are normally present in the limbus but not the cornea (Cotsarelis et al., 1989, Douvaras et al., 2013, Ksander et al., 2014, Lehrer et al., 1998, Pajoohesh-Ganji et al., 2006, Zhao et al., 2009). LRCs in the WT mouse limbus are restricted to a 100–200 μm wide region of the ocular surface (Zhao et al., 2009). If, the *Mp*/+ limbus were wider than normal, the 200 μm wide sampling box would probably include more limbal tissue when *Mp*/+ LRCs were counted. Thus, the higher counts for *Mp*/+ eyes need not imply a higher density of LRCs per area of limbus. However, as *Mp*/+ eyes have more LRCs per circumference (not just per mm of circumference), it is possible that the *Mp*/+ limbus is both wider and has a higher density of LRCs.

Many types of adult stem cells are quiescent (Li and Clevers, 2010) and lineage tracing suggests that LESCs cycle though phases of quiescence and activity (Dorà et al., 2015). Stem cells may be identified as LRCs either because they divide slowly or because they enter quiescence. The greater number of LRCs per sampling area, observed in the *Mp*/+ limbus, might suggest that the *Mp*/+ limbus contained a higher proportion of quiescent LESCs than WT. However, whether a greater proportion of quiescent LESCs results in more LRCs will depend on the balance of two opposite effects (a relative decrease in the number of cells that become labelled and an increase in the proportion of labelled cells that retain the label for 10 weeks). Evidence from X-inactivation mosaics suggests that the *Mp*/+ limbus contains more active LESC clones per mm of circumference and, overall, it seems likely that the total number of LESCs per mm of circumference is greater in *Mp*/+ than WT eyes. This is also consistent with the possibility, suggested earlier, that the limbus and LESCs occupy a wider region of the ocular surface in *Mp*/+ than WT eyes.

If the *Mp*/+ eye does have more LESCs, they seem barely able to keep up with the demand for corneal epithelial cells because the corneal epithelium is thin and loosely packed even though the *Mp*/+ cornea is greatly reduced in diameter. However, we found no convincing evidence that corneal epithelial cell proliferation was reduced or cell loss was much greater in *Mp*/+ eyes. It remains possible, though, that corneal maintenance is compromised because the *Mp*/+ LESCs function poorly. Further investigations with markers, such as ABCB5, that identify LESCs and probably early TACs (Ksander et al., 2014), or Cre-*loxP* lineage tracing (Amitai-Lange et al., 2015, Di Girolamo et al., 2015, Dorà et al., 2015) may help test the predictions that the *Mp*/+ limbus is abnormally wide and contains more LESCs than normal but they function poorly.

### 4.4 Conclusions

Together, our investigations indicate that heterozygous *Mp*/+ mice have a novel combination of corneal phenotypes. The thin, loosely packed *Mp*/+ corneal epithelium and the presence of goblet cells imply that corneal epithelial maintenance is impaired. The distribution of K19-positive and K12-positive cells and limbal blood vessels suggest either that the limbal-corneal boundary is poorly defined or the limbus is wider than normal. Indirect analyses with label-retaining cells and mosaic patterns provided no evidence that *Mp*/+ LESC numbers were depleted but suggested that they may be more numerous than usual yet function poorly. Further investigations are required to characterise the *Mp*/+ limbus more fully, test the predictions about *Mp*/+ LESCs and clarify to what extent ER stress in the corneal and/or limbal stroma contributes to the observed abnormalities of corneal epithelial morphology and maintenance.

## Conflicts of interest

None of the authors have any conflicts of interest.

## Authors’ contributions

PD, NJD, RLM and EJL performed the experiments, PD, NJD, RLM, EJL and JDW analysed the data and prepared the figures, JDW and REH designed the experiment and supervised the work, JDW and PD wrote the first draft and all authors contributed to the preparation of the final manuscript.

## References

[bib1] Ahmad S. (2012). Concise Review: limbal stem cell deficiency, dysfunction, and distress. Stem Cells Transl. Med..

[bib2] Amitai-Lange A., Altshuler A., Bubley J., Dbayat N., Tiosano B., Shalom-Feuerstein R. (2015). Lineage tracing of stem and progenitor cells of the murine corneal epithelium. Stem Cells.

[bib3] Beebe D.C., Masters B.R. (1996). Cell lineage and the differentiation of corneal epithelial cells. Investig. Ophthalmol. Vis. Sci..

[bib4] Buck R.C. (1985). Measurement of centripetal migration of normal corneal epithelial cells in the mouse. Investig. Ophthalmol. Vis. Sci..

[bib5] Chen W., Hara K., Tian Q., Zhao K., Yoshitomi T. (2007). Existence of small slow-cycling Langerhans cells in the limbal basal epithelium that express ABCG2. Exp. Eye Res..

[bib6] Collinson J.M., Chanas S.A., Hill R.E., West J.D. (2004). Corneal development, limbal stem cell function, and corneal epithelial cell migration in the *Pax6*^*+/-*^ mouse. Investig. Ophthalmol. Vis. Sci..

[bib7] Collinson J.M., Morris L., Reid A.I., Ramaesh T., Keighren M.A., Flockhart J.H., Hill R.E., Tan S.S., Ramaesh K., Dhillon B., West J.D. (2002). Clonal analysis of patterns of growth, stem cell activity, and cell movement during the development and maintenance of the murine corneal epithelium. Dev. Dyn..

[bib8] Cotsarelis G., Cheng S.Z., Dong G., Sun T.T., Lavker R.M. (1989). Existence of slow-cycling limbal epithelial basal cells that can be preferentially stimulated to proliferate: implications on epithelial stem cells. Cell..

[bib9] Davis J., Duncan M.K., Robison W.G., Piatigorsky J. (2003). Requirement for Pax6 in corneal morphogenesis: a role in adhesion. J. Cell Sci..

[bib10] Di Girolamo N., Bobba S., Raviraj V., Delic N.C., Slapetova I., Nicovich P.R., Halliday G.M., Wakefield D., Whan R., Lyons J.G. (2015). Tracing the fate of limbal epithelial progenitor cells in the murine cornea. Stem Cells.

[bib11] Dorà N.J., Hill R.E., Collinson J.M., West J.D. (2015). Lineage tracing in the adult mouse corneal epithelium supports the limbal epithelial stem cell hypothesis with intermittent periods of stem cell quiescence. Stem Cell Res..

[bib12] Douvaras P., Mort R.L., Edwards D., Ramaesh K., Dhillon B., Morley S.D., Hill R.E., West J.D. (2013). Increased corneal epithelial turnover contributes to abnormal homeostasis in the *Pax6*^*+/-*^ mouse model of aniridia. PLoS One.

[bib13] Hodson B.A., Unbekandt M., Keighren M.A., Springbett A., West J.D. (2011). Evaluation of methods for one-dimensional spatial analysis of two-dimensional patterns in mouse chimaeras. J. Anat..

[bib14] Ksander B.R., Kolovou P.E., Wilson B.J., Saab K.R., Guo Q., Ma J., McGuire S.P., Gregory M.S., Vincent W.J.B., Perez V.L., Cruz-Guilloty F., Kao W.W.Y., Call M.K., Tucker B.A., Zhan Q., Murphy G.F., Lathrop K.L., Alt C., Mortensen L.J., Lin C.P., Zieske J.D., Frank M.H., Frank N.Y. (2014). ABCB5 is a limbal stem cell gene required for corneal development and repair. Nature.

[bib15] Lehrer M.S., Sun T.T., Lavker R.M. (1998). Strategies of epithelial repair: modulation of stem cell and transit amplifying cell proliferation. J. Cell Sci..

[bib16] Li L.H., Clevers H. (2010). Coexistence of quiescent and active adult stem cells in mammals. Science.

[bib17] Morrison J.C., Fraunfelder F.W., Milne S.T., Moore C.G. (1995). Limbal microvasculature of the rat eye. Investig. Ophthalmol. Vis. Sci..

[bib18] Mort R.L. (2009). Quantitative analysis of patch patterns in mosaic tissues with ClonalTools software. J. Anat..

[bib19] Mort R.L., Bentley A.J., Martin F.L., Collinson J.M., Douvaras P., Hill R.E., Morley S.D., Fullwood N.J., West J.D. (2011). Effects of aberrant *Pax6* gene dosage on mouse corneal pathophysiology and corneal epithelial homeostasis. PLoS One.

[bib20] Mort R.L., Douvaras P., Morley S.D., Dorà N., Hill R.E., Collinson J.M., West J.D., Kubiak J.Z. (2012). Stem cells and corneal epithelial maintenance: insights from the mouse and other animal models. Results Probl Cell Differ, 55, Mouse Development: from Oocyte to Stem Cells.

[bib21] Mort R.L., Ramaesh T., Kleinjan D.A., Morley S.D., West J.D. (2009). Mosaic analysis of stem cell function and wound healing in the mouse corneal epithelium. BMC Dev. Biol..

[bib22] Nagasaki T., Zhao J. (2003). Centripetal movement of corneal epithelial cells in the normal adult mouse. Investig. Ophthalmol. Vis. Sci..

[bib23] Nakamura T., Hamuro J., Takaishi M., Simmons S., Maruyama K., Zaffalon A., Bentley A.J., Kawasaki S., Nagata-Takaoka M., Fullwood N.J., Itami S., Sano S., Ishii M., Barrandon Y., Kinoshita S. (2014). LRIG1 inhibits STAT3-dependent inflammation to maintain corneal homeostasis. J. Clin. Investig..

[bib24] Nishida K., Kinoshita S., Ohashi Y., Kuwayama Y., Yamamoto S. (1995). Ocular surface abnormalities in aniridia. Am. J. Ophthalmol..

[bib25] Pajoohesh-Ganji A., Pal-Ghosh S., Simmens S.J., Stepp M.A. (2006). Integrins in slow-cycling corneal epithelial cells at the limbus in the mouse. Stem Cells.

[bib26] Pajoohesh-Ganji A., Pal-Ghosh S., Tadvalkar G., Stepp M.A. (2012). Corneal goblet cells and their niche: implications for corneal stem cell deficiency. Stem Cells.

[bib27] Pal-Ghosh S., Pajoohesh-Ganji A., Brown M., Stepp M.A. (2004). A mouse model for the study of recurrent corneal epithelial erosions: α9β1 integrin implicated in progression of the disease. Investig. Ophthalmol. Vis. Sci..

[bib28] Phipps, E., 1964. Mp. Mouse News Letter (Private Comminication). 31, 41.

[bib29] Phipps, E., 1965. Micropinna-microphthalmia, Mp. Mouse News Letter (Private Communication). 33, 68.

[bib30] Puangsricharern V., Tseng S.C.G. (1995). Cytologic evidence of corneal diseases with limbal stem cell deficiency. Ophthalmology.

[bib31] Quinn J.C., West J.D., Hill R.E. (1996). Multiple functions for *Pax6* in mouse eye and nasal development. Genes Dev..

[bib32] Rainger J., Keighren M., Keene D.R., Charbonneau N.L., Rainger J.K., Fisher M., Mella S., Huang J.T.J., Rose L., van’t Hof R., Sakai L.Y., Jackson I.J., FitzPatrick D.R. (2013). A trans-acting protein effect causes severe eye malformation in the *Mp* mouse. PLoS Genet..

[bib33] Ramaesh T., Collinson J.M., Ramaesh K., Kaufman M.H., West J.D., Dhillon B. (2003). Corneal abnormalities in *Pax6*^*+/-*^ small eye mice mimic human aniridia-related keratopathy. Investig. Ophthalmol. Vis. Sci..

[bib34] Ramaesh T., Ramaesh K., Collinson J.M., Chanas S.A., Dhillon B., West J.D. (2005). Developmental and cellular factors underlying corneal epithelial dysgenesis in the *Pax6*^*+/-*^ mouse model of aniridia. Exp. Eye Res..

[bib35] Roach S.A. (1968). The Theory of Random Clumping.

[bib36] Sartaj R., Chee R.-I., Yang J., Wan P., Liu A., Guaiquil V., Fuchs E., Rosenblatt M.I. (2016). LIM homeobox domain 2 is required for corneal epithelial homeostasis. Stem Cells.

[bib37] Schmidt G.H., Wilkinson M.M., Ponder B.A.J. (1986). Non-random spatial arrangement of clone sizes in chimaeric retinal pigment epithelium. J. Embryol. Exp. Morph.

[bib38] Secker G.A., Daniels J.T. (2008). Corneal epithelial stem cells: deficiency and regulation. Stem Cell Rev..

[bib39] Shi S.R., Key M.E., Kalra K.L. (1991). Antigen retrieval in formalin-fixed, paraffin-embedded tissues: an enhancement method for immunohistochemical staining based on microwave oven heating of tissue sections. J. Histochem Cytochem.

[bib40] Shi Y., Tu Y., De Maria A., Mecham R.P., Bassnett S. (2013). Development, composition, and structural arrangements of the ciliary zonule of the mouse. Investig. Ophthalmol. Vis. Sci..

[bib41] Shi Y., Tu Y., Mecham R.P., Bassnett S. (2013). Ocular phenotype of *Fbn2*-null mice. Investig. Ophthalmol. Vis. Sci..

[bib42] Smith R.S., Sundberg J.P., John S.W.M., Smith R.S. (2002). The anterior segment and ocular adnexae. Systematic Evaluation of the Mouse Eye. Anatomy, Pathology and Biomethods.

[bib43] Tan S.-S., Williams E.A., Tam P.P.L. (1993). X-chromosome inactivation occurs at different times in different tissues of the post-implantation mouse embryo. Nat. Genet..

[bib44] Thoft R.A., Friend J. (1983). The X, Y, Z hypothesis of corneal epithelial maintenance. Investig. Ophthalmol. Vis. Sci..

[bib45] West J.D. (1976). Clonal development of the retinal epithelium in mouse chimaeras and X-inactivation mosaics. J. Embryol. Exp. Morphol..

[bib46] Yoshida S., Shimmura S., Kawakita T., Miyashita H., Den S., Shimazaki J., Tsubota K. (2006). Cytokeratin 15 can be used to identify the limbal phenotype in normal and diseased ocular surfaces. Investig. Ophthalmol. Vis. Sci..

[bib47] Zhang W., Zhao J., Chen L., Urbanowicz M.M., Nagasaki T. (2008). Abnormal epithelial homeostasis in the cornea of mice with a destrin deletion. Mol. Vis..

[bib48] Zhao J., Chen L., Nagasaki T. (2011). Corneal vascularization and loss of homeostatic epithelial cell movements. Investig. Ophthalmol. Vis. Sci..

[bib49] Zhao J., Mo V., Nagasaki T. (2009). Distribution of label-retaining cells in the limbal epithelium of a mouse eye. J. Histochem Cytochem.

