# Supplementary material for: Abnormal corneal epithelial maintenance in mice heterozygous for the micropinna microphthalmia mutation Mp
Source: Exp Eye Res. 2016 Aug;149:26–39. doi: 10.1016/j.exer.2016.05.021 (PMC4974241; doi:10.1016/j.exer.2016.05.021)
Supplement: Supplementary file 1 [file mmc1.pdf]

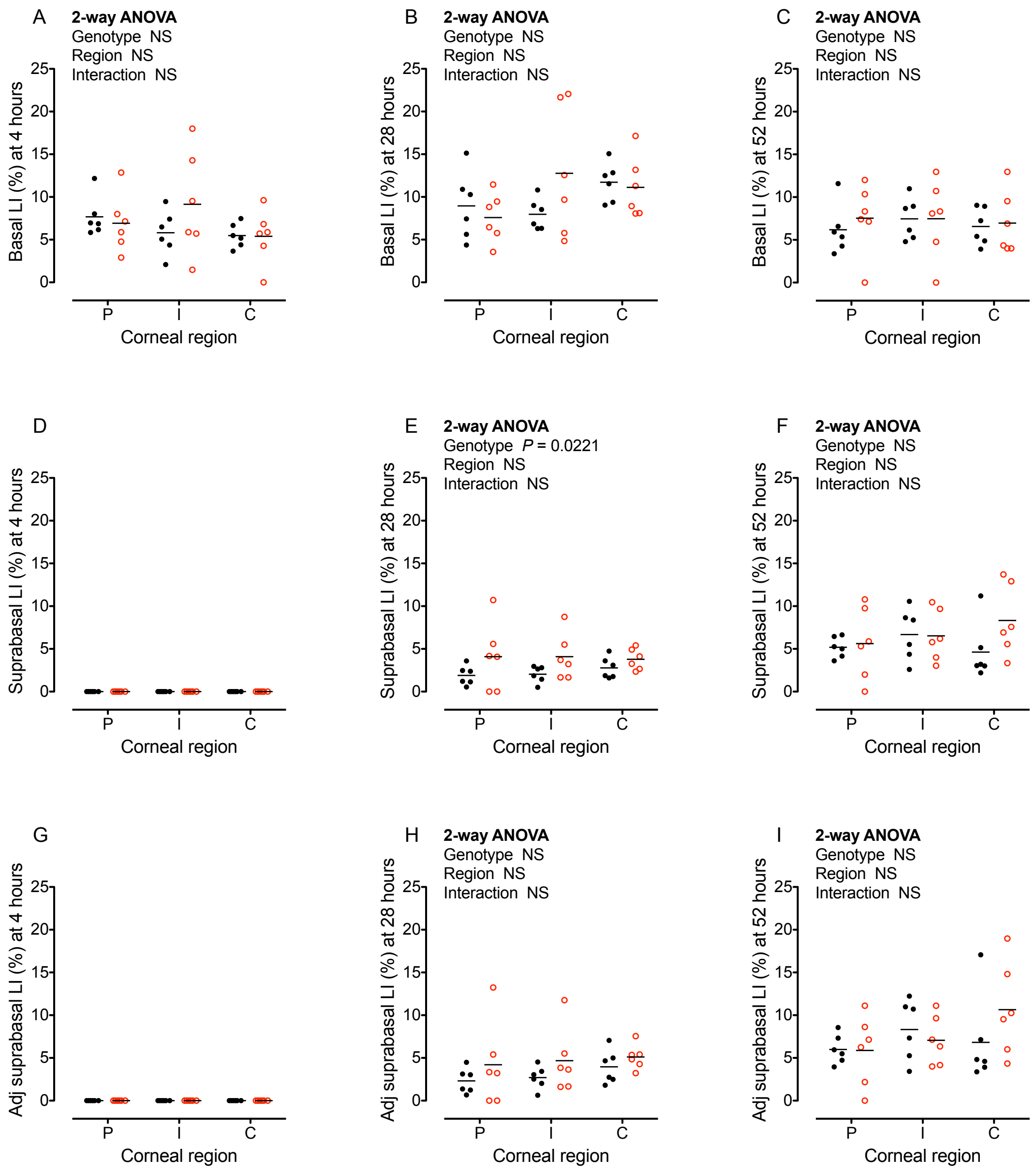

Supplementary Fig. S1. Corneal epithelial turnover in different corneal regions. Fifteen-week old mice were injected with BrdU and the percentage BrdU-positive cells (labelling index, LI) in different layers of the corneal epithelium (excluding the limbus) was determined by immunohistochemistry of mid-sections for chase periods of 4, 28 and 52 hours. Results for the whole corneal epithelium are shown in Fig. 3. (**A-C**) Basal BrdU LI (BrdU positive basal cells as a percentage of total basal cells). (**D-F**) Suprabasal BrdU LI (BrdU positive suprabasal cells as a percentage of total suprabasal cells). (**G-I**) Adjusted suprabasal BrdU LI (BrdU positive suprabasal cells as a percentage of total basal cells to allow for any differences in number of cell layers). The BrdU LI was analysed separately for the peripheral (P), intermediate (I) and central (C) regions of the cornea (excluding the limbus). No significant regional differences were identified. Genotype differences were only significant overall in (E) but pairwise comparisons between genotypes using Bonferroni post-hoc tests revealed no significant differences between genotypes for any of the three corneal regions individually. The right eyes were scored for six mice of each genotype at each of the three time points. Each point in the graphs represents one eye and the mean is shown by a horizontal bar. NS, not significant.
